# Research on the Layout Design of Auxiliary Support Modules for Suppressing Machining Chatter in Thin-Walled Beams

**DOI:** 10.3390/ma18091986

**Published:** 2025-04-27

**Authors:** Junping Feng, Yifei Gu, Zhuang Mu, Jiawei Wang, Zongyang Du, Wenbo He, Kean Aw, Yinfei Yang

**Affiliations:** 1 School of Mechanical Engineering, Jiangsu University of Technology, Changzhou 213001, China; jxfjp@jstu.edu.cn (J.F.); guyifei2022@163.com (Y.G.); 15651318505@163.com (J.W.); 18238852118@163.com (Z.D.); 2 College of Mechanical and Electrical Engineering, Nanjing University of Aeronautics & Astronautics, Nanjing 210016, China; 18896790207@163.com; 3 Nanjing Hangdian Intelligent Manufacturing Technology Co., Ltd., Nanjing 210014, China; wenbo_he@outlook.com; 4 Yangtze River Delta Intelligent Manufacturing Innovation Center, Nanjing 210014, China; 5 Department of Mechanical and Mechatronics Engineering, University of Auckland, Auckland 1010, New Zealand; k.aw@auckland.ac.nz

**Keywords:** weak-stiffness thin-walled beam, cutting chattering, auxiliary support, natural frequency, module layout

## Abstract

A well-designed clamping layout significantly enhances the dynamic stiffness of a manufacturing system, improving its stability and suppressing cutting chatter in workpieces. This paper focuses on the machining of thin-walled beams, which are prone to vibration and have low stiffness, especially under hydraulic floating clamping conditions. By analyzing the system stability domain, we propose a method to improve system stiffness through strategic design of support module layouts. Finite element dynamic simulations and modal hammer experiments were conducted to validate this approach. The results show that the proposed layout design method increases the relative central frequency by 13.49% and the relative fundamental frequency by 8.51%. These findings demonstrate a substantial improvement in the dynamic stiffness of the part-clamping system, confirming that the auxiliary support module layout design method effectively enhances system dynamic stiffness and suppresses cutting chatter.

## 1. Introduction

Thin-walled components are widely used in aerospace, nuclear power equipment, automobile manufacturing, and other fields because of their lightweight and compact structure [1]. However, thin-walled structural parts are prone to chatter during actual processing and production, affecting their processing quality [2,3]. The widespread machining chattering of large-size, thin-walled components is an urgent problem that machining technology must solve. Therefore, research on suppressing the machining chatter of thin-walled components has crucial practical significance [4,5].

Damping-based methods focus on absorbing and dissipating vibrational energy to reduce chatter. Ruttanatri P [6] proposed a design methodology for a machining chatter suppression feedback controller that combines flexible structures and cutting force models, demonstrating notable efficacy in chatter control. In order to suppress milling chattering of thin-walled workpieces, Du [7] proposed an active control method using a piezoelectric patch actuator to stabilize the whole process by designing a controller and setting the parameters at the maximum vibration position. Liu Haibo [8] developed a vibration suppression technique utilizing additional mass and eddy current damping for milling thin-walled parts. These methods often employ materials or devices with inherent damping properties to enhance machining stability.

Tool and material optimization methods aim to improve machining stability by selecting appropriate tool materials and optimizing cutting parameters. Cai [9] proposed a tool orientation optimization algorithm based on stiffness matching, and, based on the phenomenon that different deformations occur when applying forces of the same size but in different directions, he proposed the concept of maximum stiffness orientation. Zhou [10] proposed a digital twin model in which two sub-models, cutting parameter optimization and vibration detection, were established to effectively suppress the chattering of thin-walled parts in milling.

Active control methods utilize real-time adjustments and control systems to counteract vibrations. Wu Yan [11] introduced an active chatter control scheme for turning thin-walled parts using giant magnetostrictive drivers to address cutting process chatter. Shao Li [12] investigated chatter control in blade profile machining, designing a casting head sleeve tool to enhance blade blank quality and stiffness, thereby improving system stability and reducing chatter. These techniques often involve advanced control strategies and actuators to stabilize the machining process dynamically.

Intelligent monitoring and prediction methods leverage data analytics, machine learning, and sensor technologies to predict and mitigate chatter. Zheng [13] proposed a method to quickly calculate force-induced deformations using a radial basis function neural network agent model, which was constructed with a small amount of finite element simulation data, and was able to accurately predict deformations and their distribution. Wang Zhixue [14] reviewed the progress in intelligent flutter monitoring during cutting processes, emphasizing sensor selection, feature extraction, flutter recognition, and suppression. These approaches aim to enhance machining stability through proactive and informed decision-making.

Fixture and support design methods focus on stabilizing the workpiece during machining by optimizing the clamping and support structures. Liu Zhixue [15] designed specialized fixtures to optimize the machining process of thin-walled aviation casings, effectively reducing workpiece chatter and enhancing processing efficiency. Hou Junming [16] collected acceleration signals, sound signals, and surface roughness after processing vibration tests. A fitting empirical model reflecting the mapping relationship between the three has been established, and a quantitative analysis and vibration suppression evaluation method has been proposed based on the surface quality and machining accuracy of the workpiece. These strategies enhance the rigidity of the machining setup and reduce vibrations.

Despite these advancements, relatively few studies have focused on the strategic layout design of auxiliary support modules to suppress machining chatter in thin-walled components. Addressing this gap, the present study introduces a novel approach focusing on the layout design of auxiliary support modules to enhance system dynamic stiffness and suppress machining chatter. In this study, we focus on a thin-walled beam with low stiffness and propose a layout design methodology for auxiliary support modules aimed at suppressing flutter during the machining process. The effectiveness of the proposed layout design is validated through both simulation and experimental tests, demonstrating its potential to improve the machining stability and quality of thin-walled components significantly.

## 2. Auxiliary Support Module Layout Design Method

### 2.1. Mechanical Analysis of Load Vibration of Hydraulic Clamping System

**Figure 1:**
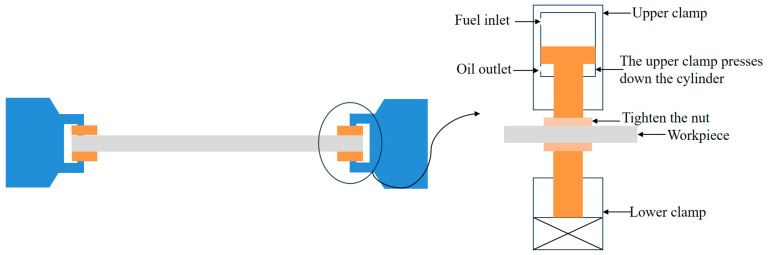
External load structure of hydraulic floating clamping method.

Most large thin-walled parts adopt the hydraulic floating clamping method. This method’s external load comprises the upper and lower clamping jaws of the floating clamping module and the workpiece in the middle clamping state. The external load structure of the simplified floating clamping method and its local magnification are shown in Figure 1.

**Figure 2:**
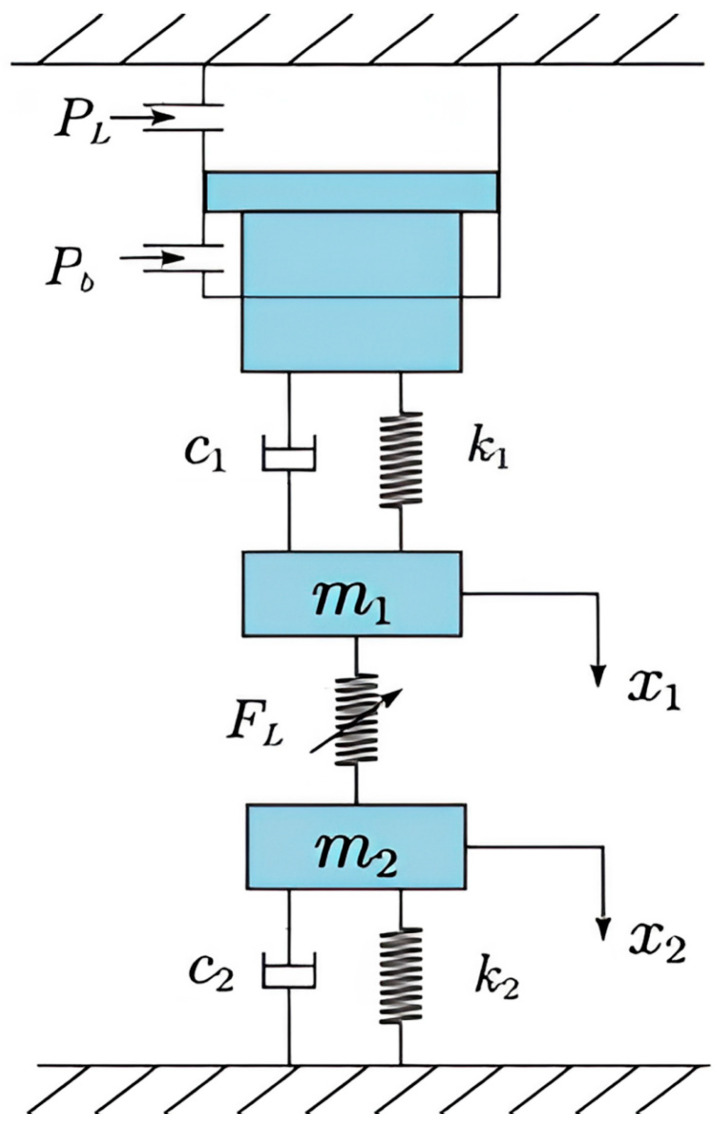
A two-degree-of-freedom mechanical model of a floating clamping method load system.

The floating clamping system can be regarded as a mechanical system composed of mass, damping, stiffness, and other factors. Based on the two-degree-of-freedom model, this paper studies the modelling method of the load system of the floating clamping method. Taking the upper clamping jaw system as one quality system and the lower clamping jaw system as another, the two-degree-of-freedom mechanical model of the load system of the floating clamping method is established, as shown in Figure 2.

In the figure, $P_{L}$ is the rodless chamber working pressure, $P_{b}$ is the rodded chamber working pressure. $m_{1}$ and $m_{2}$ are the total mass of the moving parts of the upper and lower grippers, respectively; $c_{1}$ and $c_{2}$ are the equivalent damping coefficients of the moving parts of the upper and lower grippers, respectively; $k_{1}$ and $k_{2}$ are the equivalent stiffness coefficients of the moving parts of the upper and lower grippers, respectively; $x_{1}$ and $x_{2}$ are the displacement of the upper and lower grippers, respectively; $F_{L}$ is the load force acting on the workpiece. $A_{P}$ is the piston area of the rodless cavity of the lower cylinder, and $A_{b}$ is the effective working area of the rodless cavity of the lower cylinder. According to Newton’s second law, the mechanical balance equation of the load system of the floating clamping method is as follows:

(1) $$p_{L}A_{P}-p_{b}A_{b}=m_{1}{\ddot{x}}_{1}+c_{1}{\dot{x}}_{1}+k_{1}x_{1}+F_{L}$$

(2) $$F_{L}=m_{2}{\ddot{x}}_{2}+c_{2}{\dot{x}}_{2}+k_{2}x_{2}$$

### 2.2. System Stability Analysis of Hydraulic Floating Clamping Method

According to the transfer relation derived in Section 2.1, the influence of disturbance quantity on the position closed-loop system and pressure closed-loop system of the floating clamping method is analyzed, and the stability region of the system is sought.

When the system is in balance at the operating point A, the balance equation of the hydraulic transmission part is:

(3) $$p_{LA}A_{P}-p_{b}A_{b}=m_{1}{\ddot{x}}_{1A}+c_{1}{\dot{x}}_{1A}+k_{1}x_{1A}+F_{LA}$$

In the above formula, $p_{LA}$ is the working pressure of the rodless cavity at point A, $x_{1A}$ is the displacement value of the piston rod movement to point A, and $F_{LA}$ is the load force at work point A. When the system is subjected to a small disturbance at the operating point A, the load force balance equation should be satisfied as follows:

(4) $$\left(p_{LA}+∆p_{L}\right)A_{P}-p_{b}A_{b}=m_{1}\left({\ddot{x}}_{1A}+∆\ddot{x}\right)+c_{1}\left({\dot{x}}_{1A}+∆\dot{x}\right)+k_{1}\left(x_{1A}+∆x\right)+F_{LA}$$

From the associative Equations (3) and (4) is obtained the following:

(5) $$∆p_{L}A_{P}=m_{1}∆\ddot{x}+c_{1}∆\dot{x}+k_{1}∆x$$

When the system is subjected to a small disturbance at the operating point A, the approximate equation of the disturbance flow of the hydraulic transmission part is shown in (6), and the flow continuity equation of the hydraulic cylinder is shown in (7).

(6) $$∆Q_{L}=K_{q}∆X_{v}-K_{c}∆p_{L}$$

(7) $$∆Q_{L}=A_{P}∆\dot{x}+C_{ip}∆p_{L}+\frac{V_{0}}{\beta_{e}}∆{\dot{p}}_{L}$$

where $K_{q}$ is the flow gain, $K_{c}$ is the flow-pressure coefficient, $C_{ip}$ is the leakage coefficient, $V_{0}$ is the initial volume of the control chamber, and $\beta_{e}$ is the elastic modulus of the oil.

By connecting (5)–(7) and carrying out Laplace transformation, the relation between the disturbance quantity of hydraulic cylinder load displacement Δx and the disturbance quantity of hydraulic cylinder spool Δ$x_{v}$ and the relation between the disturbance quantity of hydraulic cylinder load pressure $∆p_{L}$ and the disturbance quantity of the hydraulic cylinder spool Δ$x_{v}$ can be obtained:

(8) $$∆x=\frac{A_{p}}{s\left[\frac{V_{0}m_{1}}{\beta_{e}}s^{2}+\left(K_{ce}m_{1}+\frac{V_{0}c_{1}}{\beta_{e}}\right)s+K_{ce}c_{1}+\frac{V_{0}k_{1}}{\beta_{e}}+A_{p}^{2}\right]+k_{1}K_{ce}}K_{q}∆x_{v}$$

(9) $$∆P_{L}=\frac{1}{s\left(\frac{A_{p}^{2}}{m_{1}s^{2}+c_{1}s+k_{1}}+\frac{V_{0}}{\beta_{e}}\right)+K_{ce}}K_{q}∆x_{v}$$

where $K_{ce}$ is the total flow–pressure coefficient, $K_{ce}=C_{ip}+K_{c}$. $K_{1}$ is the equivalent stiffness coefficient of the moving parts of the upper claw system, and $K_{q}$ is the flow gain.

The transfer function between the disturbance quantity of hydraulic cylinder load displacement Δx and the disturbance quantity of hydraulic cylinder spool displacement Δ$x_{v}$ and the disturbance quantity of hydraulic cylinder load pressure $∆p_{L}$ and the disturbance quantity of hydraulic cylinder Δ$x_{v}$ is summarized as

(10) $$G_{1}\left(s\right)=\frac{A_{p}}{s\left[\frac{V_{0}m_{1}}{\beta_{e}}s^{2}+\left(K_{ce}m_{1}+\frac{V_{0}c_{1}}{\beta_{e}}\right)s+K_{ce}c_{1}+\frac{V_{0}k_{1}}{\beta_{e}}+A_{p}^{2}\right]+k_{1}K_{ce}}$$

(11) $$G_{2}\left(s\right)=\frac{1}{s\left(\frac{A_{p}^{2}}{m_{1}s^{2}+c_{1}s+k_{1}}+\frac{V_{0}}{\beta_{e}}\right)+K_{ce}}$$

where $G_{1}\left(s\right)$ is the transfer function of the perturbation amount of hydraulic cylinder load displacement and the perturbation amount of hydraulic cylinder spool displacement; $G_{2}\left(s\right)$ is the transfer function of the perturbation amount of hydraulic cylinder load pressure and the perturbation amount of hydraulic cylinder displacement. When the whole floating clamping system works in the position closed-loop system mode with the help of sensor feedback and PC calculation control, the relation between the disturbance amount of hydraulic cylinder spool displacement Δ$x_{v}$ and the disturbance amount of hydraulic cylinder load displacement Δx is as follows:

(12) $$∆X_{v}=G_{c}\left(s\right)G_{x}\left(s\right)∆x$$

where $G_{c}(s)$ is the transfer function of the PID regulating system of the upper computer PC, and $G_{x}(s)$ is the transfer function of the displacement sensor. Let $G_{3}(s)=G_{c}(s)G_{x}(s)$; then $G_{3}(s)$ is the nonlinear transfer function between the disturbance of the hydraulic cylinder spool displacement Δ$x_{v}$ and the disturbance of the hydraulic cylinder load displacement Δx.

Similarly, when the whole floating clamping system works in the pressure closed-loop system mode with the help of pressure feedback and the calculation control of PC, the relation between the disturbance amount of the hydraulic cylinder spool Δ$x_{v}$ and the disturbance amount $∆p_{L}$ of the hydraulic cylinder load pressure is as follows:

(13) $$∆X_{v}=G_{c}\left(s\right)G_{p}\left(s\right)∆p_{L}$$

where $G_{p}(s)$ is the pressure sensor transfer function. Let $G_{4}(s)=G_{c}(s)G_{p}(s)$; then $G_{4}(s)$ is the nonlinear transfer function between the hydraulic cylinder spool displacement disturbance Δ$x_{v}$ and the hydraulic cylinder load pressure disturbance $∆p_{L}$.

In this paper, the Popov theorem is used as the theoretical criterion of system stability to find the stability region of the system. According to Popov’s theorem, when the nonlinear characteristic function f[e(t)] of a nonlinear closed-loop feedback system satisfies the following formula, the system is globally asymptotically stable at its equilibrium point:

(14) $$f\left(0\right)=0\;\;\;0\leq \frac{f\left[e\left(t\right)\right]}{e\left(t\right)}\leq P$$

The Popov criterion is used to study the stability of the position closed loop system and find the stability region of the position closed loop system. Therefore, in the transfer function $G_{1}\left(s\right)$, s=jw gives $G_{1}\left(jw\right)=Re_{1}\left(w\right)+jIm_{1}\left(w\right)$, and its modified frequency characteristic function is derived as follows:

(15) $$G_{1}^{*}\left(jw\right)=X_{1}\left(w\right)+jY_{1}\left(w\right)$$

(16) $$X_{1}\left(w\right)=Re_{1}\left(w\right)Y_{1}\left(w\right)=wIm_{1}\left(w\right)$$

Solve the coordinates of the intersection of $G_{1}^{*}\left(jw\right)$ curve with the real axis, that is, the critical point of the Popov criterion, and let its coordinates be (−$P_{1}^{-1}$,0). According to (15) and (16), the *x*-coordinate value of the critical point is obtained as follows:

(17) $$X_{1}\left(w^{*}\right)=\frac{A_{p}V_{0}m_{1}\beta_{e}}{\beta_{e}\left(k_{ce}m_{1}\beta_{e}+V_{0}c_{1}\right){(k}_{ce}c_{1}+A_{p}^{2})+V_{0}^{2}k_{1}c_{1}}$$

From the definition of the Popov line, it can be deduced that ${-P_{1}^{-1}=X}_{1}\left(w^{*}\right)$, from which we can see that the value of $P_{1}$, in accordance with Equation (14), is

(18) $$P_{1}=-\frac{1}{X_{1}\left(\omega^{*}\right)}=\frac{\beta_{e}\left(K_{ce}m_{1}\beta_{e}+V_{0}c_{1}\right)\left(K_{ce}c_{1}+A_{p}^{2}\right)+V_{0}^{2}k_{1}c_{1}}{A_{p}V_{0}m_{1}\beta_{e}}$$

According to Popov’s theorem, when the nonlinear characteristic function $f_{1}\left(∆e\right)=G_{3}(s)K_{q}∆e$ of the position closed-loop system satisfies Equation (14), the system will tend to be stable. Thus, the stability region of the position closed-loop system is derived as follows:

(19) $$G_{3}\left(s\right)K_{q}\leq \frac{\beta_{e}\left(K_{ce}m_{1}\beta_{e}+V_{0}c_{1}\right)\left(K_{ce}c_{1}+A_{p}^{2}\right)+V_{0}^{2}k_{1}c_{1}}{A_{p}V_{0}m_{1}\beta_{e}}$$

Similarly, the modified frequency characteristic function $G_{2}^{*}\left(iw\right)$ of the pressure closed-loop system is derived, and the intersection point of the $G_{2}^{*}\left(iw\right)$ curve with the real axis is analyzed, that is, the horizontal coordinate value of the critical point, and then the stability region of the pressure closed-loop system is obtained as follows:

(20) $$G_{4}\left(S\right)K_{q}\leq \frac{2c_{1}{k_{1}V}_{0}^{2}}{\beta_{e}\left[V_{0}c_{1}^{2}-m_{1}\beta_{e}A_{P}^{2}-\sqrt{{\left(V_{0}c_{1}^{2}-m_{1}\beta_{e}A_{p}^{2}\right)}^{2}-4m_{1}k_{1}V_{0}^{2}c_{1}^{2}}\right]}-\frac{c_{1}V_{0}}{m_{1}\beta_{e}}-K_{ce}$$

From a qualitative point of view, the effect of nonlinear damping force will affect the amplitude of the system, which can be expressed by the nonlinear damping effect coefficient β. The increase of β will reduce the amplitude of the system, and the increase of β will narrow the resonance band of the system and reduce the unstable region of the system, thus effectively improving the stability of the system.

In the floating clamping hydraulic system adopted in this study, the damping mainly comes from the friction damping between the cylinder wall and the piston and between the load workpiece and the clamping nut of the upper and lower jaw. At the same time, the elastic rubber expansion head of the auxiliary support module can also greatly improve the damping of the system, reduce the amplitude of the system, inhibit the vibration of the workpiece during processing, and ensure the stability of the system. In order to improve the dynamic rigidity of the process system and improve the vibration of the structural parts in the process, the damping coefficient of the system can be changed by adding damping elements in the floating clamping system, so as to increase the natural frequency of the system and improve the dynamic stiffness of the system. Therefore, when the auxiliary support module layout strategy is given to suppress the machining vibration of thin wall beams with weak stiffness, the structure flutter can be suppressed by adding auxiliary support devices in response to the deformation of the web where the amplitude of the web is larger.

### 2.3. An Auxiliary Support Module Layout Strategy for Vibration Suppression of Thin-Walled Beams with Weak Stiffness

**Figure 3:**
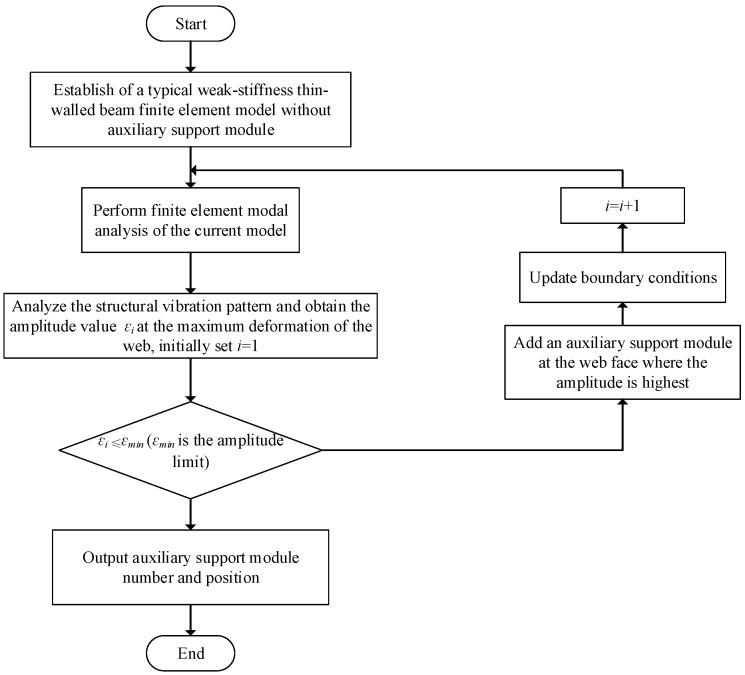
Layout design flowchart of the auxiliary support module.

In the process of milling thin-walled beams with weak stiffness, the machining chatter of the web will affect the machining quality of the parts. Therefore, in order to better meet the machining requirements of the workpiece, this study conducted a modal simulation analysis of the thin-walled beam-fixture system with the purpose of suppressing the machining chatter of typical thin-walled beams, and obtained the amplitude of each key node on the web. The auxiliary support module is added at the maximum amplitude of the web, and the auxiliary support is analyzed and added in a cycle until the amplitude value $\varepsilon_{i}$ at the maximum deformation of the web is less than or equal to the minimum amplitude limit $\varepsilon_{min}$. At the end of the cycle, the position and quantity of the auxiliary support module are output. The layout design process of the auxiliary support module is shown in Figure 3 below.

Step 1: The finite element model of the weak-stiffness thin-walled beam without adding an auxiliary support module is established according to the confirmed optimization results of the clamping layout of the floating clamping module.

Step 2: The modal analysis of the current model is carried out in the finite element software to obtain the vibration mode of the weak-stiffness thin-walled beam and the amplitude at the key point in this state.

Step 3: The structural mode is analyzed and the amplitude $\varepsilon_{i}$ at the maximum deformation of the web is obtained.

Step 4: Determine whether the amplitude value $\varepsilon_{i}$ at the maximum deformation is less than or equal to the minimum amplitude limit $\varepsilon_{min}$.

Step 5: If less than, output the number and position of auxiliary support modules, that is, the layout scheme of auxiliary support modules to suppress the machining vibration of weak-stiffness thin-walled beams; if greater than, add auxiliary support modules to the maximum amplitude of web plate.

Step 6: Update the boundary conditions and go back to Step 2.

Through the above layout design process, the final layout result of the auxiliary support module at the position of the web is obtained. The forced vibration analysis of the thin-walled beam under the final layout is carried out in the test section to verify the feasibility of the layout design of the proposed auxiliary support module.

## 3. Dynamic Simulation and Experimental Verification of Auxiliary Support Module Layout Design

### 3.1. The Establishment of Simulation Model

**Figure 4:**
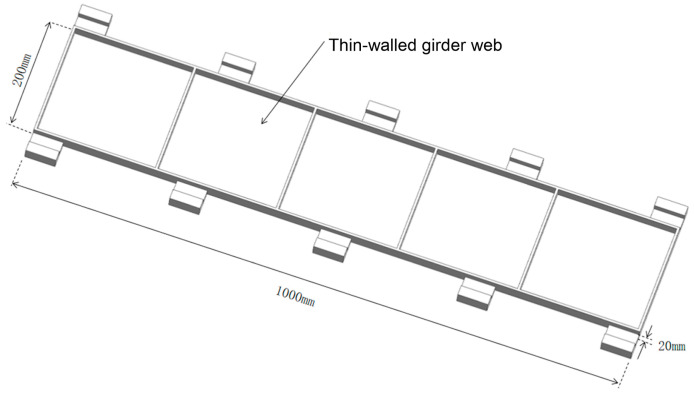
Thin-walled beam structural parts.

The modal analysis and forced vibration analysis of the typical beam structure were carried out by the finite element method, and the dynamic characteristics of the beam structure under different local clamping layout were analyzed, so as to obtain the auxiliary support module layout which can increase the stiffness of the thin-walled parts to meet the machining accuracy requirements. During the finite element simulation, the beam-type structural part is taken as the research object, as shown in Figure 4. The workpiece size is 1000 mm × 200 mm × 20 mm, the groove depth is 16 mm, the thickness of the side wall and the web are 4 mm, the structure is symmetrical, and the material is 7050-T7451 aluminum alloy.

**Figure 5:**
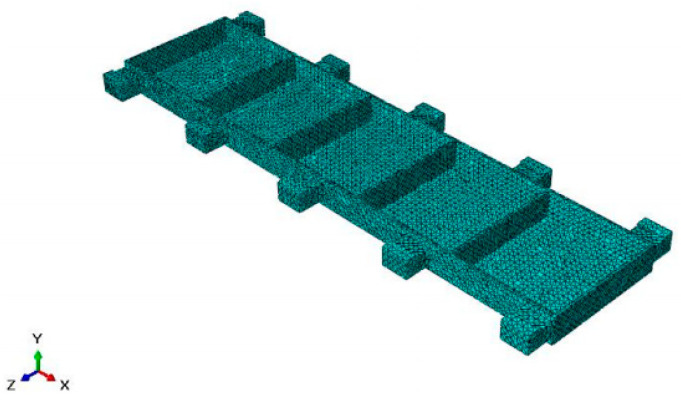
Mesh division of finite element model.

Firstly, three-dimensional modeling of the beam structure is carried out in the modeling software, which is imported into the simulation software, endows the material properties, which are shown in the Table 1 below, and completes the section assignment. The frequency analysis under the linear perturbation analysis step is selected in the analysis step module, and the characteristic modes are extracted according to the method. Since it is a thin-walled model, the approximate global size of 10 mm was selected when the grid was divided, and 71,913 grids were divided in tetrahedral mesh form, as shown in Figure 5.

In order to verify the effectiveness of the method to suppress the machining flutter of thin-walled beams by adding auxiliary support modules, the modal simulation of parts under different auxiliary support module layout states is required. The simulation results are analyzed and compared. The first three module layout states are shown as follows: Layout state 1 indicates layout optimization, but no auxiliary support is added, and the maximum amplitude $\varepsilon_{1}$ is obtained. Layout state 2 represents the identification of the danger point with the largest amplitude in the simulation result of layout state 1, and the placement of auxiliary support modules here to obtain the maximum amplitude $\varepsilon_{2}$. Layout state 3 represents the identification of the danger point with the largest amplitude in the simulation results of layout state 2, and the addition of auxiliary support modules here to obtain the maximum amplitude value $\varepsilon_{3}$.

### 3.2. Analysis of the Simulation Results

**Figure 6:**
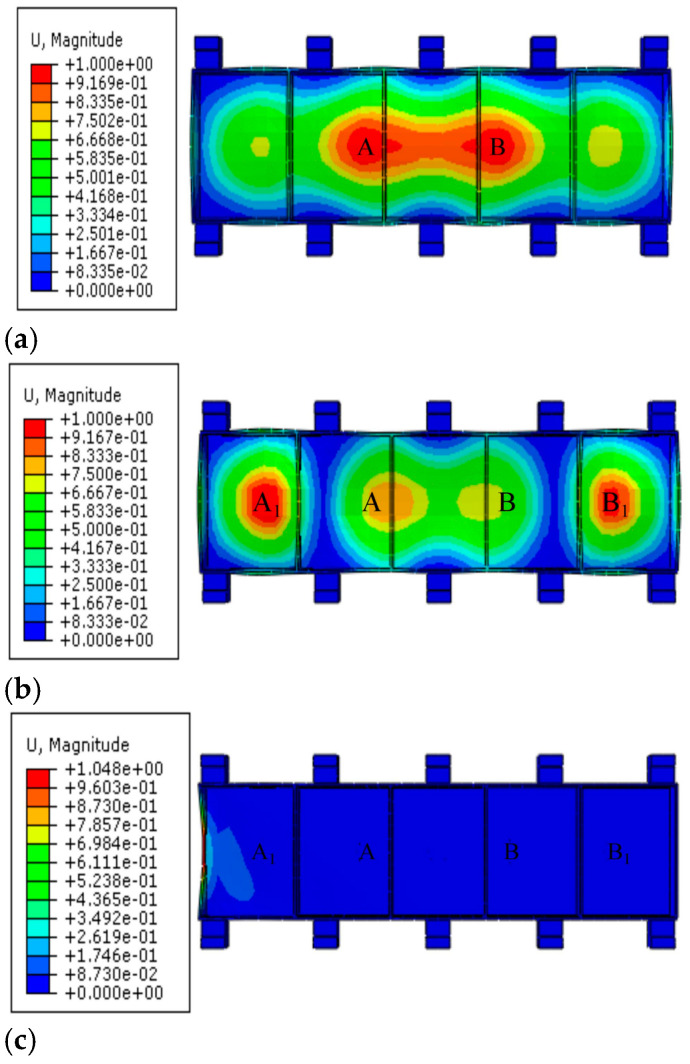
Simulation results of modal analysis of thin-walled parts. (**a**) Layout status 1; (**b**) Layout status 2; (**c**) Layout status 3.

The comparison cloud image of the modal simulation results of the parts under the first three layout states is shown in Figure 6. In layout state 1, that is, without an auxiliary support module, the amplitudes of danger points A and B are the largest, the displacement $\varepsilon_{1}$ at the maximum amplitude is 1.00008 mm, and $\varepsilon_{1}>\varepsilon_{min}$. In layout state 2, that is, when a pair of auxiliary support modules are added at positions A and B, the maximum displacement response of dangerous points A and B is only 0.0493 mm, and the maximum displacement is greatly reduced. In this case, the maximum deformation danger points are A1 and B1, the maximum displacement $\varepsilon_{2}$ is 1.00003 mm, and $\varepsilon_{2}>\varepsilon_{min}$. Continuing to add a pair of auxiliary support modules at positions A1 and B1 to obtain layout status 3, the maximum displacement of danger points A1 and B1 is reduced by 0.299 mm. The vibration of the dangerous point is improved, which proves that the auxiliary support module layout strategy described in this paper is beneficial in restraining the machining vibration of the weak-stiffness thin-walled beam.

The natural frequencies of the first six modes of the thin-walled parts-clamping system under three layout states are shown in Table 2.

By comparison, it can be seen that, according to the layout strategy of the auxiliary support module for suppressing the machining vibration of the weak-stiffness thin-walled beam described in this paper, after the auxiliary support module is continuously arranged at the dangerous point where the web amplitude is large, the natural frequencies of the first six modes of the parts-clamping system continue to increase, indicating that the rigidity of the system is constantly improved and that the stiffness of the system is improved.

**Figure 7:**
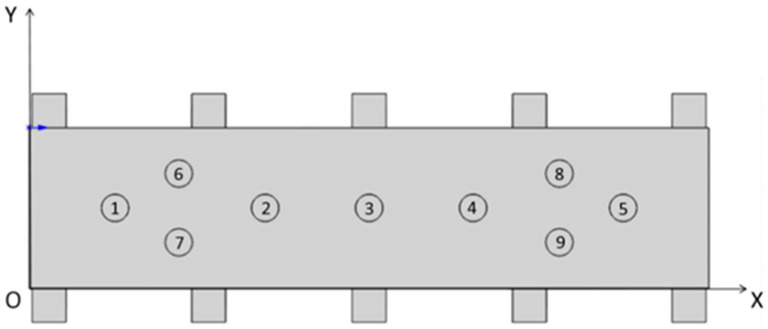
Final layout design of the auxiliary support module.

According to the above methods, the auxiliary support module’s layout designs for increasing the thin-walled beam’s stiffness were carried out in turn, and the final optimal layout results are shown in Figure 7. A floating auxiliary support module on the bottom of the web is added at the following coordinates: ① (126.25, 117.5) ② (347, 117.5) ③ (500, 117.5) ④ (653, 117.5) ⑤ (873.75, 117.5) ⑥ (220.25, 167.9) ⑦ (220.25, 67.1) ⑧ (779.75, 167.9) ⑨ (779.75, 167.9).

**Figure 8:**
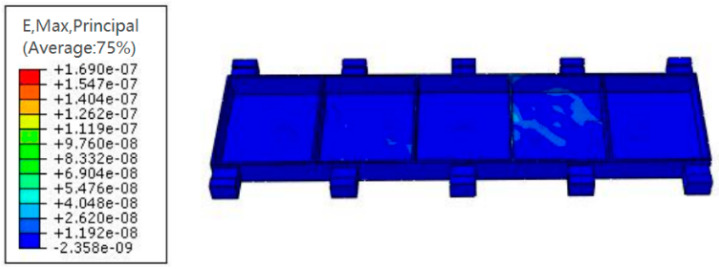
Transient strain results of the last time increment step of the part’s forced vibration.

**Figure 9:**
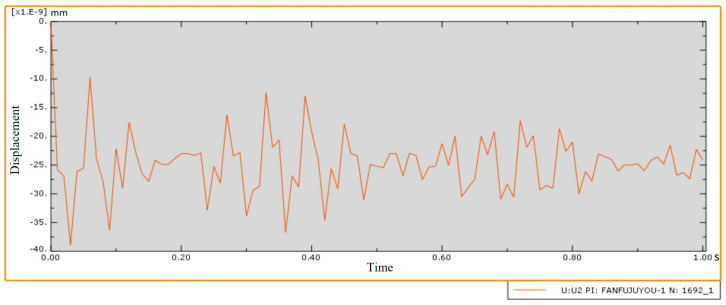
Y-direction displacement curve of the center point of web structure with time.

Dynamic response simulation analysis was carried out on the thin-walled beam model under the layout of the auxiliary support module to simulate its forced vibration under the action of milling force, and the model strain was used to determine whether the parts met the processing requirements. The results are shown in Figure 8. It can be seen from the figure that the internal transient strain of the thin-walled beam under the final auxiliary support layout meets the machining requirements when it is subjected to an external load caused by milling force. In addition, the center point of the web structure is a dangerous point prone to large transient displacement during forced vibration of such beam structural parts. The change curve of Y-displacement over time is shown in Figure 9. The transient displacement of the center point of the web structure is very small during the whole time range of forced vibration, which can meet the processing requirements.

## 4. Modal Hammering Experiment

**Figure 10:**
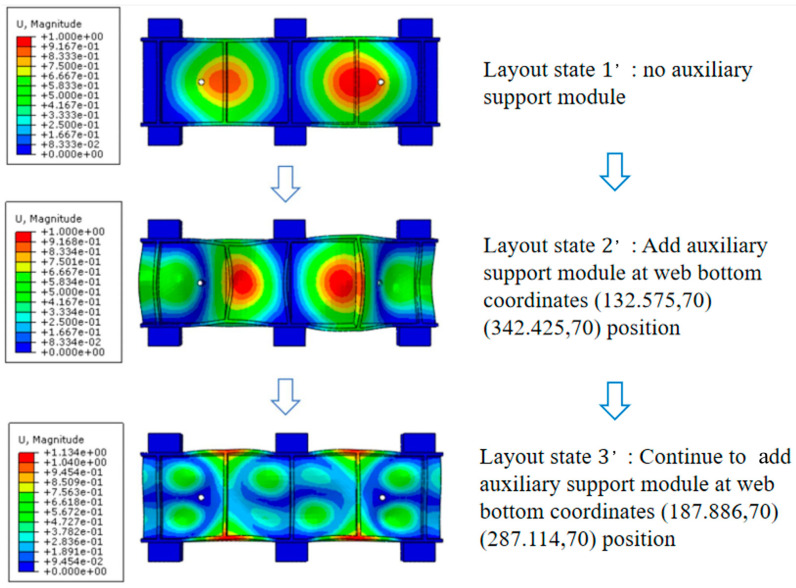
Layout state of modal hammer test.

The modal hammer test verifies the improvement of the dynamic stiffness of the thin-walled beam-clamping system by the auxiliary support module layout design method described in this paper. Considering the cost of the experiment and the constraints of the site and machine tool, according to the existing experimental conditions, the size of the beam test parts was selected as 475 mm × 140 mm × 20 mm, the thickness of the side wall as 5 mm, and the thickness of the web as 3 mm. Based on this, a finite element model was established to determine the structural vibration modes and amplitudes at key points of the web under different layout states of the auxiliary support modules through modal analysis and simulation. Auxiliary support modules were added at dangerous points with large amplitudes on the web, and the above process was iterated to obtain the auxiliary support module layout for restraining the machining vibration of weak-stiffness thin-walled beams, as shown in Figure 10.

Through the above layout design method of adding auxiliary support modules, was is finally determined to add four auxiliary support modules under the web; the position coordinates are (132.575, 70), (342.425, 70), (187.886, 70), and (287.114, 70).

The natural frequencies of the first six modes of the thin-walled piece-clamping system before and after optimized layout are shown in Table 3. It can be seen from the natural frequency data in the table that after adding auxiliary support modules successively, the natural frequency of each mode before and after the thin-walled beam-clamping system continues to increase, and the stiffness of the system also continues to increase from the relationship between the natural frequency and the stiffness.

**Figure 11:**
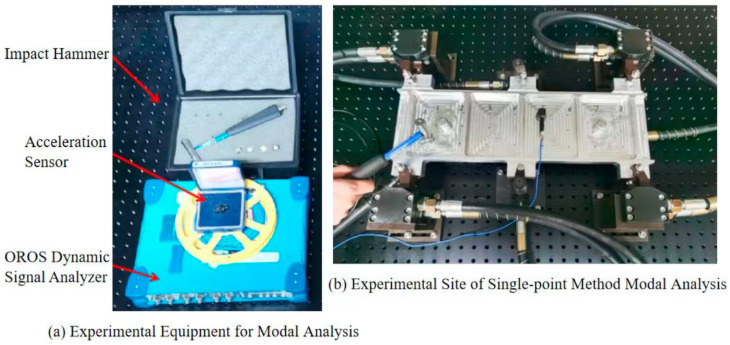
Experimental setup for single point method for modal analysis.

The experimental site of this modal experiment is shown in Figure 11. The beam structure parts after milling are taken, and four floating clamping modules and two angle modules are used for floating clamping to achieve a floating clamping environment. The modal hammer experiment is carried out by a single-point method; that is, the position of the sensor is fixed at a certain point on the web structure, and the striking position of the modal hammer is constantly changed. The clamping state before and after the layout design of the auxiliary support module is respectively modally hammered; the time domain waveform of the excitation signal and the corresponding signal are respectively collected by the modal impact hammer and the three-direction acceleration sensor; the signal spectrum is obtained and analyzed by the modal signal analysis software; and the transfer function is obtained by the data processing software for data processing and output.

**Figure 12:**
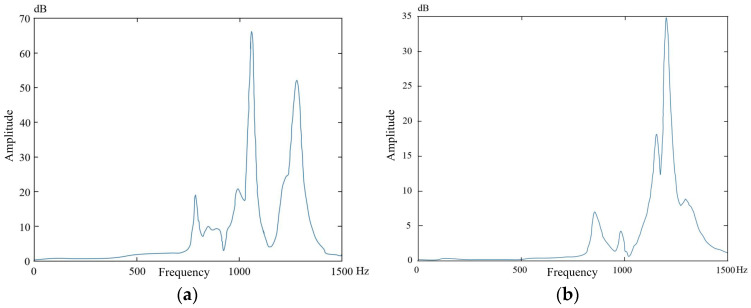
Amplitude frequency diagrams (**a**) before and (**b**) after auxiliary support module layout design.

The vibration amplitude-frequency comparison of the thin-walled parts-mounting system before and after the auxiliary support module layout design is obtained through the modal hammer experiment, as shown in Figure 12.

Based on the comparative analysis of Figure 12a,b, after implementing the layout design of the auxiliary support module to increase the stiffness of thin-walled parts, the following observations were made:

The main frequency of the parts-mounting system increased from 1060 Hz to 1203 Hz, marking an increase of 143 Hz, corresponding to a relative increase of 13.49%.

The fundamental frequency increased from 787 Hz to 854 Hz, an increase of 67 Hz, corresponding to a relative increase of 8.51%. The maximum magnitude was reduced from 66 db to 34 db, a relative reduction of 48.48%.

These changes indicate that the stiffness of the parts-clamping system has been enhanced. As a result, the machining requirements can now be met more effectively, which verifies the effectiveness of the auxiliary support module layout design method. This design approach successfully increases the stiffness of thin-walled parts, thereby suppressing the flutter of parts during machining.

## 5. Conclusions

This research focuses on the layout design method of an auxiliary support module aimed at enhancing the rigidity of thin-walled parts in the system. The study begins with a dynamic analysis of the system under floating clamping conditions. A vibration mechanics model of the cutting load is established to explore the stability domain of the system’s dynamic characteristics. Based on this analysis, a layout strategy for the auxiliary support module is proposed to suppress vibration during thin-walled beam machining.

Finite element dynamic simulation and modal hammering experiments were conducted to validate the proposed design. The results demonstrate that the optimal design of the auxiliary support module significantly enhances the system’s performance. Specifically, the natural frequency of the sixth-order mode increased by 62.8%, the main frequency of the parts-clamping system rose by 13.49%, and the fundamental frequency improved by 8.51%. The intrinsic frequencies all increased, which proves that the system stiffness was increased and that the effect of improving the system stiffness was achieved. These findings confirm that the proposed design method effectively increases system stiffness and mitigates machining chatter.

## Figures and Tables

**Table 1:** Properties of 7050-T7451 aluminum alloy materials.

Density ρ (kg/m^3^)	Young’s Modulus E (Gpa)	Poisson’s Ratio v
2800	70.3	0.33

**Table 2:** Modal natural frequencies of the parts-clamping system in three auxiliary support module layouts.

Degree	Inherent Frequency (Hz)		
	Layout Status 1	Layout Status 2	Layout Status 3
1	1938.2	2238.4	2290.0
2	2080.6	2256.5	2895.8
3	2155.3	2328.4	2911.9
4	2180.5	3066.9	3066.2
5	2704.8	3251.5	3363.2
6	2955.1	3322.9	3420.8

**Table 3:** Modal natural frequencies of the parts-clamping system under different auxiliary support module layout states.

Degree	Inherent Frequency (Hz)		
	Layout Status 1	Layout Status 2	Layout Status 3
1	2115.4	2955.3	3170.2
2	2133.0	3503.5	3705.5
3	3512.6	3585.2	3844.7
4	3570.9	3712.1	4252.7
5	3642.9	4277.7	4692.9
6	3681.8	4926.7	5995.0

## Data Availability

The original contributions presented in this study are included in the article. Further inquiries can be directed to the corresponding author.
